# A patient with spastic paralysis finally diagnosed as V180I genetic Creutzfeldt-Jakob disease 9 years after onset

**DOI:** 10.1080/19336896.2020.1823179

**Published:** 2020-09-17

**Authors:** Taichi Nomura, Ikuko Iwata, Ryoji Naganuma, Masaaki Matsushima, Katsuya Satoh, Tetsuyuki Kitamoto, Ichiro Yabe

**Affiliations:** a Department of Neurology, Faculty of Medicine and Graduate School of Medicine, Hokkaido University, Sapporo, Hokkaido, Japan; b Department of Molecular Microbiology and Immunology, Graduate School of Biomedical Sciences Nagasaki University, Nagasaki, Japan; c Center for Prion Diseases, Tohoku University Graduate School of Medicine, Miyagi, Japan

**Keywords:** Creutzfeldt-Jakob disease, spastic paralysis, V180i genetic Creutzfeldt-Jakob disease, Gerstmann-Sträussler-Scheinker disease, cortical dysfunction

## Abstract

Genetic Creutzfeldt-Jakob disease (gCJD) with a mutation in codon 180 of the prion protein gene (V180I gCJD) is the most common form of gCJD in Japan, but only a few cases have been reported in Europe and the United States. It is clinically characterized by occurring in the elderly and presenting as slowly progressive dementia, although it generally shows less cerebellar and pyramidal symptoms than sporadic CJD. Here, we report a patient with V180I gCJD who initially presented with slowly progressive spastic paralysis with neither cerebrospinal fluid (CSF) nor magnetic resonance imaging (MRI) abnormalities. His symptoms progressed gradually, and after 9 years, he displayed features more typical of CJD. Diffusion-weighted MRI revealed high-intensity signals in the cortical gyrus, and there was a marked increase of 14-3-3 protein and total tau protein in the CSF, but he was negative for the real-time quaking-induced conversion assay. Although the time course was more consistent with Gerstmann-Sträussler-Scheinker disease than CJD, genetic testing revealed V180I gCJD. This is the first report of a patient with V180I gCJD who initially presented with spastic paralysis, and also the first to reveal that it took 9 years from disease onset for cortical dysfunction to develop and for MRI and CSF abnormalities to be detectable. In conclusion, we should screen for V180I gCJD in elderly patients presenting with slowly progressive spastic paralysis.

## Introduction

Creutzfeldt-Jakob disease (CJD) is a rare, fatal, and transmissible neurodegenerative disease. In CJD, genetic CJD (gCJD) is differentiated by mutation of the prion protein gene, which accounts for 10.2% and 16.7% of cases in Europe and Japan, respectively. In Japan, unlike in other countries, the most frequent mutation occurs in codon 180 of the prion protein (PrP) gene (V180I gCJD) [1,2]. In fact, in a 10-year surveillance of CJD in Japan, Nozaki et al. reported that of 1222 patients diagnosed as CJD, 17.7% were classified as gCJD, and 41.2% of gCJD patients had the V180I mutation [1].

The clinical features of V180I gCJD are: 1) older onset age (late 70s); 2) slower progression; 3) characteristic clinical symptoms such as more frequent higher cortical dysfunction, less visual or cerebellar symptoms, less myoclonic jerks, and less pyramidal signs compared with sporadic (sCJD); 4) a lower positive rate of brain-specific proteins such as neuron-specific enolase, 14-3-3 protein, total tau protein, and real-time quaking-induced conversion (RT-QUIC) assay in cerebrospinal fluid (CSF); and 5) absence of periodic synchronous discharges in electroencephalography throughout the clinical course [1–7].

Despite being a genetic disease, patients with V180I CJD rarely have a family history, and only 5.9% of patients have a family history of dementia [2]. Although the initial symptoms in V180I gCJD vary in each case, most cases present with cortical dysfunction such as disorientation, aphasia, and apraxia [2,4–8]. Therefore, patients with gCJD are often misdiagnosed with dementia such as Alzheimer’s disease. However, due to its transmissible nature, it is very important to diagnose CJD. Here, we report a case with V180I gCJD who was difficult to diagnose because his initial symptom was spastic paralysis, and it took 9 years for him to begin to show progressive dementia.

## Clinical summary

In early 2010, a 67-year-old Japanese man noticed clumsiness of his right leg. He had never undergone brain surgery. His sister was diagnosed with amyotrophic lateral sclerosis (ALS), but there was no family history of spastic paralysis. His sister developed dysphasia and dysarthria at 62 years of age, but she did not show spasticity. Electromyography (EMG) revealed neurogenic changes of her upper and lower limbs. She died at 4 years after onset and pathological dissection was not performed. His father died from tuberculosis at 58 years of age and his mother died from stroke at 68 years of age, but they did not have the symptoms of a neurodegenerative disease at the time of death.

**Figure 1.**
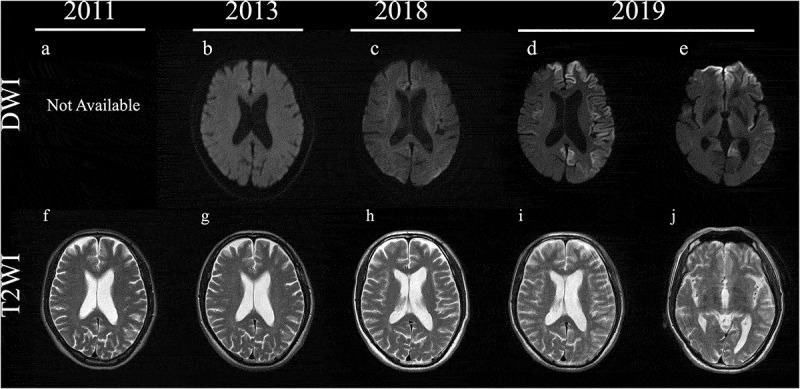
Magnetic resonance imaging (MRI) findings in diffusion-weighted images (DWI) and T2-weighted images (T2WI) in the brain from 2011 to 2019. MRI examinations were normal in DWI and T2WI from 2011 to 2018 (a–c, g–h). In 2019, right dominant cortical high-intensity signals, except in the occipital lobe, in DWI (d) and abnormal swelling in T2WI (i) were detected. The corpus callosum and caudate were normal in DWI and T2WI (c,j).

**Figure 2.**
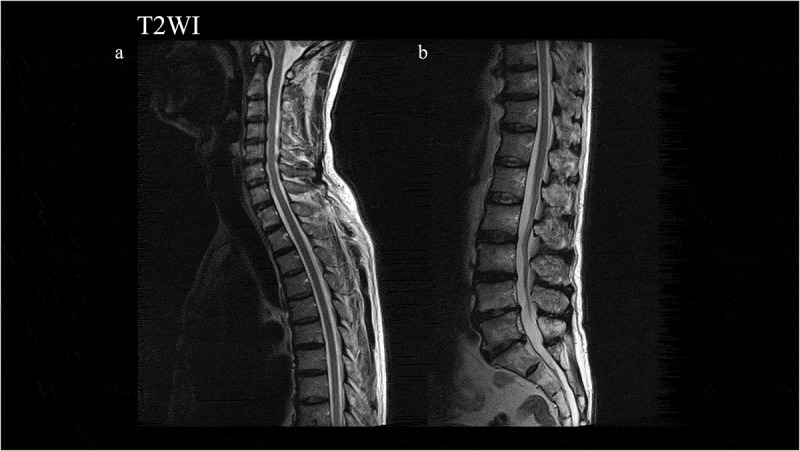
Magnetic resonance imaging findings in T2-weighted images (T2WI) in the spinal cord. Normal findings were observed.

He gradually felt awkwardness of his right leg. In November 2011, he was admitted to our hospital and showed weakness of the right lower extremity with spastic paralysis (Manual Muscle Test scores of 4 for the right iliopsoas and 4 for the right tibialis anterior), increased deep tendon reflex of the right patellar and Achilles tendons, right Babinski’s sign, increased muscle tone and spasticity in his right lower limb, and difficulty with walking. He did not demonstrate any signs of ataxia and apraxia. CSF examination and MRI of the brain and spinal cord were normal (Figures 1(a,f) and 2), including an absence of anti-human T-cell lymphotropic virus type 1 (HTLV1) antibodies. EMG was performed on the right biceps brachii, first dorsal interossei, rectus femoris, tibial anterior, and tongue, but neither neurogenic nor myogenic changes were observed.

In November 2013, he experienced difficulties with gait and standing from bed. At the second hospitalization, the right lower extremity showed worsening spasticity and a limping gait, although there was almost no change in muscle strength. The upper and left lower extremities showed no obvious motor deficits at this time. MRI findings of the brain and spinal cord were not changed (Figure 1(b,g)). A nerve conduction study revealed no abnormalities, and neither neurogenic nor myogenic signs were observed on EMG. A repeat CSF examination was still normal.

At the April 2014 outpatient visit, he showed bilateral right dominant spasticity. He had difficulty standing on his own from June 2014 and needed a walker in 2015. As of the August 2016 visit, the spasticity had spread to the upper extremities besides the bilateral lower limbs in a right dominant manner, and the lower extremity muscles became weaker (Manual Muscle Test scores of 2/2 for the right/left iliopsoas and 2/2 for the right/left quadriceps femoris). In December 2017, he displayed dysarthria and dysphagia and showed right dominant weakness in his upper limb. From August 2018, although he could perform daily activities such as reading a newspaper and managing money, he had been using a wheelchair and showed consistent right dominant spasticity and weakness. Brain MRI in the same month did not reveal any noteworthy findings (Figure 1(c,h)).

**Figure 3.**
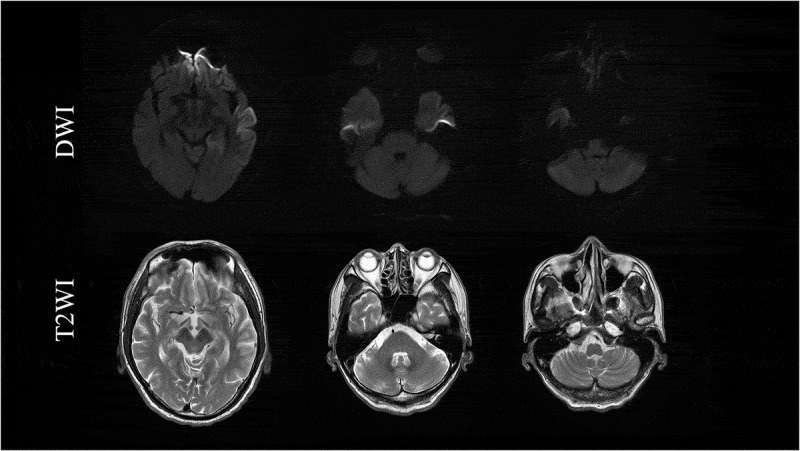
Magnetic resonance imaging findings in T2-weighted images (T2WI) and diffusion-weighted images (DWI) in the brainstem. Normal findings were observed.

**Figure 4.**
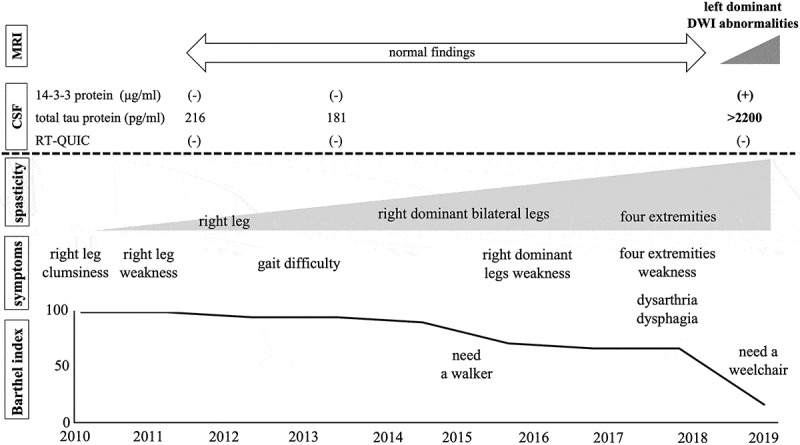
Clinical course. The patient complained of right leg clumsiness in 2010. While he revealed gradually progressive right dominant spastic paralysis, magnetic resonance imaging (MRI) and cerebrospinal fluid (CSF) examinations were normal. In 2018, he displayed spasticity and weakness of all four extremities, dysarthria, and dysphagia. MRI showed bilateral left dominant gyriform hyperintense signals on diffusion-weighted imaging. CSF examination revealed the presence of 14-3-3 protein and t-tau protein, but he was negative for the real-time quaking-induced conversion assay.

In August 2019, he showed urinary incontinence and needed assistance with all aspects of his daily life and was thus suspected of dementia. At the time of his third admission in October 2019, he displayed aphasia, apraxia, gegenhalten, bilateral lower limb grasping reflex, and bilateral Chaddock’s sign. He could not understand the doctor’s orders, indicating the rapid progression of dementia from the assessment in August, and thus we could not perform any cognitive tests. MRI revealed bilateral left dominant gyriform hyperintense signals on diffusion-weighted imaging (DWI) (Figure 1(d,e,i,j)). Although he showed bulbar symptoms, MRI findings of the brainstem were normal (Figure 3). CSF examination showed the presence of 14-3-3 protein (>500 μg/mL) and t-tau protein (>2200 pg/mL), but the RT-QUIC assay was negative. Due to dysarthria and aphasia since 2017, it was difficult to determine when dementia had begun. While myoclonus and periodic synchronous discharges were not observed throughout his clinical course, the MRI findings and CSF abnormalities implied CJD as the differential diagnosis. Genetic testing revealed *PRNP* V180I, codon 129 M/M, and he was diagnosed as V180I gCJD. In December 2019, he developed akinetic mutism. After diagnosis with V180I gCJD, we examined CSF samples taken in 2011 and 2013, but these samples were negative for 14-3-3 protein, t-tau protein, and the RT-QUIC assay (Figure 4).

## Discussion

Here, we report a patient with V180I gCJD who initially presented with spastic paralysis. He did not show any signs of CJD in the first 8 years after onset; however, at 9 years after onset, we finally detected characteristic MRI and CSF abnormalities.

This case suggests that the development of spastic paralysis in this patient might have been a very early manifestation of the V180I mutation, prior to the more typical rapidly progressive dementia. Since blood and CSF examinations were normal in the first 3 years after onset, and MRI was normal in the first 8 years after onset, we excluded myelitis, infarction, and infection such as HTLV1. It is possible he developed familial ALS or spastic paralysis accompanied with gCJD, as he never received a genetic test for familial ALS or hereditary spastic paralysis. However, since the EMG findings were normal, the present patient did not display any lower motor neuron signs. Thus, it is unlikely that he had ALS in addition to V180I gCJD. As for hereditary spastic paralysis, since the estimated prevalence is 0.2 cases per 100,000 individuals in Japan [9,10] and the patient revealed no family history of spastic paralysis, it is unlikely he had autosomal dominant hereditary spastic paralysis. Although we could not completely exclude the possibility of autosomal recessive hereditary spastic paralysis without a genetic test, it is rarer than the autosomal dominant form in Japan and also unlikely to have been present [10]. Moreover, he did not show a thin corpus callosum and spinal cord in MRI. Therefore, from the clinical observations including MRI findings, it is unlikely that the patient had hereditary spastic paralysis. In 7 patients with spastic paralysis, Yamada et al. reported the presence of homozygous changes at codon 219 (Glu to Lys) of the PrP gene in 1 patient, but none of these patients had the V180I mutation, and the influence of the homozygous changes was not determined [11]. In CJD, pyramidal signs are one of the necessary diagnostic criteria, but there is a subtype in which spasticity is extremely prominent [12–15]. In most cases with V180I gCJD, the initial symptoms are related to cortical dysfunction such as disorientation, dementia, and aphasia [2,4–6,8,16,17]. Quina et al. reported that pyramidal signs are less frequent in patients with V180I gCJD than in those with sCJD [2]. Although patients with sCJD and other types of gCJD, including Gerstmann-Sträussler-Scheinker disease, can present with spastic paralysis as the initial symptom [12–15,18,19], to the best of our knowledge, there are no reports of patients with V180I gCJD who initially presented with upper or lower motor neuron symptoms. Furthermore, the present patient showed a slowly progressive time course and he developed cortical dysfunction at 9 years after disease onset. Such a prolonged prodromal phase is untypical in patients with CJD, since their disease course usually progresses rapidly [2]. Some V180I gCJD patients survive for a long period of time [8,20], and Hayashi et al. suggested that this was probably due to tube feeding and very mild brainstem involvement [20]. However, these long-term survival patients also show rapid progression from disease onset, unlike in the present case. Thus, this case is worthy of reporting.

In addition, while the case’s parents did not live long enough to express V180I gCJD, he had a sister who was diagnosed with ALS and died at 4 years after onset. Some CJD patients, including V180I gCJD patients, present with similar symptoms to ALS or frontotemporal lobar degeneration and *vice versa* [20–22]. In the present case, although his sister did not receive DW-MRI of the brain, cognitive function tests, or genetic testing for gCJD, it is possible that her motor neuron symptoms may have been the result of V180I gCJD.

CSF testing revealed that 14-3-3 protein, total tau protein, and RT-QUIC assay were normal, at least for the first 3 years from onset, but became positive after 9 years, except for the RT-QUIC assay. Patients with V180I gCJD often have a low positive rate for 14-3-3 protein, total tau protein, and RT-QUIC assay in CSF examinations [2], which is consistent with the findings of the present case, who was still negative for the RT-QUIC assay even after 14-3-3 protein and total tau protein were detected. Amano et al. reported a patient with gCJD V180I who was negative for the RT-QUIC assay throughout the course of the disease [3]. Quina et al. reported that 67.9% of patients with V180I gCJD are positive for the RT-QUIC assay, while 90% of those with sCJD are positive [2]. The RT-QUIC assay is a highly sensitive test to detect abnormal prion proteins in CSF [23]. In patients with V180I gCJD, many pathological reports suggest that immunoreactivity to abnormal PrP is very weak [4,5,8,24–26]. Low levels of PrP accumulation in the brain could explain the low positive rate for the RT-QUIC assay. Moreover, these pathological findings could also explain the lower neurotoxicity of PrP and longer disease duration of V180I gCJD [8].

In the present study, the patient showed normal MRI findings and he was able to perform daily activities for the first 8 years after onset. After presenting with cortical dysfunction, abnormal DWI findings were detected. Almost all V180I gCJD patients present with MRI abnormalities such as a swollen cerebral cortex in T2-weighted images and cortical hyperintense signals in DWI [1,2,5–7,27]. Some reports suggest that the abnormal cortical high-intensity signals in DWI may reflect spongiform changes, especially various-sized and nonconfluent vacuoles, in the cerebral cortex of patients with V180I gCJD [4,25,28]. Indeed, most V180I gCJD patients with cortical high-intensity signals show cortical dysfunction [1,2,5–7,27]. Furthermore, a patient with V210I gCJD, whose initial symptom was spastic paralysis, did not show any abnormal MRI findings and only showed atrophy of the corticospinal tract in diffusion tensor imaging [12]. These findings support the idea that MRI may be normal in V180I CJD before cortical dysfunction appears. Therefore, although we did not determine the exact time when cognitive dysfunction started, it is possible that cognitive impairment started along with the appearance of abnormal features in DWI.

In conclusion, we report a patient with V180I gCJD whose initial symptom was spastic paralysis, and it took 9 years for cortical dysfunction to develop, similar to the time course of Gerstmann-Sträussler-Scheinker disease. This case suggests that some V180I gCJD patients may have spastic paralysis in the prolonged prodromal phase. Since CJD is a transmissible disease, it is important to diagnose it properly by regular MRI scans and prevent its transmission to others. Therefore, we should screen for V180I gCJD when patients present with spastic paralysis, even if the patient is old, does not have a family history, or their clinical course progresses slowly.

## Geolocation information

Japan, Asia.

## References

[1] Nozaki I, Hamaguchi T, Sanjo N, et al. Prospective 10-year surveillance of human prion diseases in Japan. Brain. 2010;133(10):3043–3057. 20855418 10.1093/brain/awq216

[2] Quina T, Sanjo N, Hizume M, et al. Clinical features of genetic Creutzfeldt-Jakob disease with V180I mutation in the prion protein gene. BMJ Open. 2014;16(5):e004968. 10.1136/bmjopen-2014-004968 PMC4025468 24838726

[3] Amano Y, Kimura N, Hanaoka T, et al. Creutzfeldt-Jakob Disease with a prion protein gene codon 180 mutation presenting asymmetric cortical high-intensity on magnetic resonance imaging. Prion. 2015;9(1):29–33. 25730397 10.1080/19336896.2015.1017703 PMC4601385

[4] Iwasaki Y, Mori K, Ito M, et al. An autopsy case of Creutzfeldt-Jakob disease with a prion protein gene codon 180 mutation presenting with pathological laughing and an exaggerated startle reaction. Neuropathology. 2017;37(6):575–581. 28703419 10.1111/neup.12399

[5] Iwasaki Y, Mori K, Ito M, et al. An autopsied case of V180I Creutzfeldt-Jakob disease presenting with panencephalopathic-type pathology and a characteristic prion protein type. Neuropathology. 2011;31(5):540–548. 21269331 10.1111/j.1440-1789.2010.01192.x

[6] Iwasaki Y, Mori K, Ito M, et al. A case of V180I genetic Creutzfeldt-Jakob disease presenting with conspicuous facial mimicry. Prion. 2019;13(1):151–155. 31387445 10.1080/19336896.2019.1651181 PMC6746545

[7] Jin K, Shiga Y, Shibuya S, et al. Clinical features of Creutzfeldt-Jakob disease with V180I mutation. Neurology. 2004;62(3):502–505. 14872044 10.1212/01.wnl.0000106954.54011.80

[8] Akagi A, Iwasaki Y, Mimuro M, et al. Pathological progression of genetic Creutzfeldt-Jakob disease with a PrP V180I mutation. Prion. 2018;12(1):54–62. 29264994 10.1080/19336896.2017.1414130 PMC5871029

[9] Tsuji S, Onodera O, Goto J, et al. Sporadic ataxias in Japan—a population-based epidemiological study. Cerebellum. 2008;7(2):189–197. 18418674 10.1007/s12311-008-0028-x

[10] Koh K, Ishiura H, Tsuji S, et al. JASPAC: Japan spastic paraplegia research consortium. Brain Sci. 2018;8(8):153. 10.3390/brainsci8080153 PMC6119894 30104498

[11] Yamada M, Satoh S, Sodeyama N, et al. Spastic paraparesis and mutations in the prion protein gene. J Neurol Sci. 1995;134(1–2):215–216. 8747872 10.1016/0022-510x(95)00250-4

[12] Conte F, Giordano A, Tortora F, et al. Early-onset spastic paraparesis as presenting sign of familial Creutzfeldt-Jakob disease. Parkinsonism Relat Disord. 2015;21(12):1479–1480. 26578040 10.1016/j.parkreldis.2015.10.004

[13] Geevasinga N, Simon NG, Collins M, et al. Sporadic Creutzfeldt-Jakob disease presenting as spastic paraparesis. Eur J Neurol. 2013;20(5):e73–4. 23577611 10.1111/ene.12116

[14] Mancuso M, Siciliano G, Capellari S, et al. Creutzfeldt-Jakob disease with E200K PRNP mutation: a case report and revision of the literature. Neurol Sci. 2009;30(5):417–420. 19597763 10.1007/s10072-009-0118-7

[15] Masullo C, Bizzarro A, Guglielmi V, et al. An atypical phenotype of CJD associated with the E200K mutation in the prion protein gene. Neurol Sci. 2010;31(6):837–839. 20730466 10.1007/s10072-010-0388-0

[16] Deguchi K, Takamiya M, Deguchi S, et al. Spreading brain lesions in a familial Creutzfeldt-Jakob disease with V180I mutation over 4 years. BMC Neurol. 2012;12:144. 23176099 10.1186/1471-2377-12-144 PMC3527175

[17] Terasawa Y, Fujita K, Izumi Y, et al. Early detection of familial Creutzfeldt-Jakob disease on diffusion-weighted imaging before symptom onset. J Neurol Sci. 2012;319(1–2):130–132. 22640903 10.1016/j.jns.2012.04.004

[18] Collins S, McLean CA, Masters CL. Gerstmann-Sträussler-Scheinker syndrome, fatal familial insomnia, and kuru: a review of these less common human transmissible spongiform encephalopathies. J Clin Neurosci. 2001;8(5):387–397. 11535002 10.1054/jocn.2001.0919

[19] Ishizawa K, Mitsufuji T, Shioda K, et al. An autopsy report of three kindred in a Gerstmann-Sträussler-Scheinker disease P105L family with a special reference to prion protein, tau, and beta-amyloid. Brain Behav. 2018;8(10):e01117. 30240140 10.1002/brb3.1117 PMC6192393

[20] Hayashi Y, Iwasaki Y, Waza M, et al. Clinicopathological findings of a long-term survivor of V180I genetic Creutzfeldt-Jakob disease. Prion. 2020;14(1):109–117. 32178563 10.1080/19336896.2020.1739603 PMC7153845

[21] Hayashi Y, Iwasaki Y, Takekoshi A, et al. Anautopsy-verified case of FTLD-TDP type A with upper motor neuron-predominant motor neuron disease mimicking MM2-thalamic-type sporadic Creutzfeldt-Jakob disease. Prion. 2016;10(6):492–501. 27929803 10.1080/19336896.2016.1243192 PMC5161298

[22] Yaguchi H, Takeuchi A, Horiuchi K, et al. Amyotrophic lateral sclerosis with frontotemporal dementia (ALS-FTD) syndrome as a phenotype of Creutzfeldt-Jakob disease (CJD)? A case report. J Neurol Sci. 2017;372:444–446. 27838004 10.1016/j.jns.2016.10.038

[23] Atarashi R, Satoh K, Sano K, et al. Ultrasensitive human prion detection in cerebrospinal fluids by real-time quaking-induced conversion. Nat Med. 2011;17(2):175–178. 21278748 10.1038/nm.2294

[24] Iwasaki Y. Creutzfeldt-Jakob disease. Neuropathology. 2017;37(2):174–188. 28028861 10.1111/neup.12355

[25] Iwasaki Y, Kato H, Ando T, et al. Autopsy case of V180I genetic Creutzfeldt-Jakob disease presenting with early disease pathology. Neuropathology. 2018;38(6):638–645. 30216556 10.1111/neup.12516

[26] Kobayashi S, Saito Y, Maki T, et al. Cortical propagation of Creutzfeldt-Jakob disease with codon 180 mutation. Clin Neurol Neurosurg. 2010;112(6):520–523. 20409635 10.1016/j.clineuro.2010.03.015

[27] Yoshida H, Terada S, Ishizu H, et al. An autopsy case of Creutzfeldt-Jakob disease with a V180I mutation of the PrP gene and alzheimer-type pathology. Neuropatholody. 2010;30(2):159–164. 10.1111/j.1440-1789.2009.01048.x 19703264

[28] Matsukura K, Satoh K, Shirabe S, et al. Familial Creutzfeldt-Jakob disease with a V180I mutation: comparative analysis with pathological findings and diffusion-weighted images. Dement Geriatr Cogn Disord. 2009;28(6):550–557. 20051687 10.1159/000254842 PMC2837892

